# Reconstruction of the gastric cancer microenvironment after neoadjuvant chemotherapy by longitudinal single-cell sequencing

**DOI:** 10.1186/s12967-022-03792-y

**Published:** 2022-12-06

**Authors:** Yingtai Chen, Jianhua Yin, Lulu Zhao, Guangyu Zhou, Shichen Dong, Yueming Zhang, Penghui Niu, Hu Ren, Tianjiao Zheng, Juan Yan, Wenbin Li, Peiqin Ma, Cuijuan Zhang, Chen Wei, George Church, Guibo Li, Dongbing Zhao

**Affiliations:** 1 National Cancer Center/National Clinical Research Center for Cancer/Cancer Hospital, Chinese Academy of Medical Sciences and Peking Union Medical College, 17 Panjiayuan Nanli, Beijing, 100021 China; 2 BGI-Shenzhen, Shenzhen, 518083 Guangdong China; 3 BGl College & Henan Institute of Medical and Pharmaceutical Sciences, Zhengzhou University, Zhengzhou, 450001 Henan China; 4 BGI-Henan, Xinxiang, 453000 Henan China; 5 Department of Genetics, Harvard Medical School, Boston, MA 02115 USA; 6 Department of Molecular and Cellular Biology, Harvard University, Cambridge, MA 02138 USA; 7 BGI Education Center, University of Chinese Academy of Sciences, Shenzhen, 518083 China; 8 Wyss Institute for Biologically Inspired Engineering, Harvard University, Boston, MA 02115 USA; 9 Shenzhen Key Laboratory of Single-Cell Omics, BGI Shenzhen, Shenzhen, 518120 Guangdong China

**Keywords:** Neoadjuvant chemotherapy (NACT), Gastric cancer, Single-cell RNA sequencing

## Abstract

**Background:**

Little is known on the tumor microenvironment (TME) response after neoadjuvant chemotherapy (NACT) in gastric cancer on the molecular level.

**Methods:**

Here, we profiled 33,589 cell transcriptomes in 14 samples from 11 gastric cancer patients (4 pre-treatment samples, 4 post-treatment samples and 3 pre-post pairs) using single-cell RNA sequencing (scRNA-seq) to generate the cell atlas. The ligand-receptor-based intercellular communication networks of the single cells were also characterized before and after NACT.

**Results:**

Compered to pre-treatment samples, CD4+ T cells (P = 0.018) and CD8+ T cells (P = 0.010) of post-treatment samples were significantly decreased, while endothelial cells and fibroblasts were increased (P = 0.034 and P = 0.005, respectively). No significant difference observed with respect to CD4+ Tregs cells, cycling T cells, B cells, plasma cells, macrophages, monocytes, dendritic cells, and mast cells (P > 0.05). In the unsupervised nonnegative matrix factorization (NMF) analysis, we revealed that there were three transcriptional programs (NMF1, NMF2 and NMF3) shared among these samples. Compared to pre-treatment samples, signature score of NMF1 was significantly downregulated after treatment (P = 0.009), while the NMF2 signature was significantly upregulated after treatment (P = 0.013). The downregulated NMF1 and upregulated NMF2 signatures were both associated with improved overall survival outcomes based on The Cancer Genome Atlas (TCGA) database. Additionally, proangiogenic pathways were activated in tumor and endothelial cells after treatment, indicating that NACT triggers vascular remodeling by cancer cells together with stromal cells.

**Conclusions:**

In conclusion, our study provided transcriptional profiles of TME between pre-treatment and post-treatment for in-depth understanding on the mechanisms of NACT in gastric cancer and empowering the development of potential optimized therapy procedures and novel drugs.

**Supplementary Information:**

The online version contains supplementary material available at 10.1186/s12967-022-03792-y.

## Introduction

Gastric cancer is the fifth most fatal cancer type around the world [1]. Currently, curative surgical resection with perioperative chemotherapy is the principal treatment strategy for gastric cancer [2]. Preoperative chemotherapy, also called neoadjuvant chemotherapy (NACT), aims to improve overall survival (OS) by downstaging tumor, reducing risk of local relapses, eradicating micrometastatic disease, and increasing curative surgical resection rate [3]. Clinical trials have shown that NACT improves the prognosis of patients with gastric cancer [4–6], however, little is known on the tumor microenvironment (TME) response after NACT in gastric cancer on the molecular level.

TME is composed of various cell types and extracellular components that surround tumor cells and is nourished by a vascular network [7]. Cancer progression and metastasis are influenced by the TME, which might in turn change after NACT, affecting subsequent treatment therapies in breast cancer [8]. Thus, a deeper understanding of the changes in cell composition after NACT could offer insights to aid in the development of further treatments following NACT that exploit therapeutic vulnerabilities of the TME to control gastric cancer progression. To date, some studies examined changes in the TME with gastric cancer following NACT, however, these analyses only focused on immunohistochemical analysis level [9, 10]. A recent published study [11] targeted on NACT showed the characterization of molecular features in gastric cancer patients correlated with response to neoadjuvant chemotherapy, however, it’s limited on whole-genome, whole-exome and RNA sequencing.

Recent advancement in single-cell RNA sequencing (scRNA-seq) has allowed researchers to uncover new cellular mechanism in many fields, including cancer and immunology, and has provided a more precise understanding on transcriptional features at the level of individual cell in an unbiased manner [12]. In this study, we aimed to investigate the impacts of NACT on tumor cells and TME in patients with gastric cancer, providing the possibility for the next treatment therapies. Thus, we performed scRNA-seq based on pre-treatment and post-treatment samples from 11 gastric cancer patients in order to better understand the treatment effect in gastric cancer patients on TME and shed some light on improving the current NACT and the following therapeutic procedure.

## Methods

### Patient recruitment and ethical approval

Eleven patients with histologically confirmed gastric adenocarcinoma were enrolled in this study. All tissue samples were collected at the Center for Cancer/Cancer Hospital, Chinese Academy of Medical Sciences and Peking Union Medical College from 2017 to 2018. A total of 14 tissue samples were collected from these patients through biopsy and/or surgery, and clinicopathological characteristics were in Additional file 1: Table S1. Among the samples, 7 were pretreatment samples and 7 were post-treatment samples. Three patients contributed both pre-treatment and post-treatment samples (3 pairs of pre- and post-treatment samples). Written informed consent was obtained from all participants enrolled in this study, and ethical approval was obtained from the following institutional review boards in accordance with the Declaration of Helsinki: National Cancer Center/National Clinical Research Center for Cancer/Cancer Hospital, Chinese Academy of Medical Sciences and Peking Union Medical College (approval number: 17-156/1412; date issued: 2017-09-14).

### Preparation of single-cell suspensions

The single-cell suspensions of gastric cancer samples were prepared according to protocols previously described [13].

### Droplet-based scRNA-seq

Chromium Single-cell 3'-Library, Gel Bead & Multiplex Kit and Chip Kit (10 × Genomics, Pleasanton, CA, USA) were used to generated according to the manufacturer’s instructions. Libraries were sequenced on a BGISEQ-500.

### Processing of scRNA-seq data

Cell Ranger (version 2.0.0) was used to process raw scRNA-seq data. The transcripts were aligned to the human reference genome (GRCh38, v3.0.0, from 10X Genomics). Then, the gene expression matrices for all tumor samples were combined in R (version 3.4.1) and converted to a Seurat object using the Seurat R package (version 3.0.2) [14]. Quality filtering was performed to remove cells with < 201 or > 9000 expressed genes or greater than 20% unique molecular identifiers (UMIs) derived from the mitochondrial genome.

### Clustering and cell type identification

Clustering and cell type identification was performed as described previously [13]. Briefly, 2000 highly variable genes were selected for downstream analysis. The number of principal components was estimated by the Elbow plot. FindClusters function of the Seurat package was used for data clustering, with a K parameter of 30 and the default setting for all other parameters.

Major cell types were annotated based on the average expression of selected gene sets: epithelial cells (EPCAM, KRT19, and KRT18), myeloid cells (CD68, CSF1R, and CD68), T cells (CD2, CD3D, and CD3E), B cells (CD79A, CD19, and MS4A1), endothelial cells (CLDN5, PECAM1, and VWF), mast cells (TPSAB1, TPSB2, and KIT) and fibroblasts (COL1A1, COL3A1, and DCN). To identify subclusters of immune cells and stromal cells, cells were further clustered using canonical correlation analysis (the RunMultiCCA function) [15]. Then, based on the expression of the corresponding selected gene set, the sub-clusters were annotated.

### Copy number variation (CNV) analysis

In order to distinguish between malignant and non-malignant epithelial cells, we used inferCNV (version 0.1) to analyze CNV in epithelial cells in scRNA-seq data, as previously described [16, 17]. Then, we carried out hierarchical clustering on the CNV spectra of stromal cells and epithelial cells in each of the 14 tumor samples; among the clusters containing stromal cells, we identified the non-malignant epithelial cells in the tumor samples, while the other cells in the cluster that have the entire chromosome deletion or expansion are identified as malignant cells.

### Identification of tumor cell signatures

To analyze the intratumor signatures of tumor cells, we used LIGER (0.4.2) to identify repeated signatures in different tumor samples. LIGER uses integral NMF (iNMF) to identify metagenes shared by different data sets [18]. We set the number of factors to 3 and thus obtained 3 NMF signatures. For each NMF signature, the top 100 genes with the highest NMF score (excluding ribosomal genes and noncoding genes) were identified as the characteristic gene set of that NMF. The overall expression of the gene sets of the three NMF signatures was calculated and is displayed on the t-SNE map of tumor cells.

### Pathway activation analysis

Pathway activation analysis was performed using GSVA software (version 1.26.0) [19]. The pathways used in this analysis was from molecular signature database [20]. And R package ‘limma’ was used to compare GSVA score of pre-treatment and post-treatment samples.

### Cell–cell interaction analysis

To investigate cell–cell interaction between different cell types in gastric tumor microenvironment, we performed cell–cell ligand-receptor analysis using CellPhoneDB (version 1.1.0) [21]. The single-cell transcriptomic data of annotated tumor cells, plasma cells, B cells, CD4+ T cells, CD4+ Treg cells, CD8+ T cells, cycling T cells, DC3, DC2, endothelial cells, fibroblasts, myofibroblasts, mast cells, monocytes, and macrophages were used for this cell–cell interaction analysis.

### DEG analysis

We identified genes that were differentially expressed pretreatment and post-treatment within each cell type by using “Find Markers” in Seurat. Briefly, for each cell type, only genes that met the following criteria were considered differently expressed genes (DEGs): (1) Adjusted P-value < 0.05; (2) absolute ln fold change (FC) > 0.25; and (3) detection in at least 25% of the pretreatment or post-treatment cells evaluated. The expression of individual genes as well as the up- and downregulated genes of each cell type are displayed with violin diagrams and volcano diagrams, respectively.

### Gene Ontology analysis

Gene Ontology analysis of NMF signatures was performed using Metascape software, which is available online (http://metascape.org) [22]. Genes were uploaded to the website, and the expression analysis option was selected.

### Survival analysis

To test whether the NMF program predicted OS, we computed the average (mean) expression of the signature in the stomach adenocarcinoma (STAD) cohorts. The cohorts were downloaded from TCGA Data Portal (https://gdc-portal.nci.nih.gov/). All gastric cancers analyzed in this study were untreated primary lesions (N = 371). To visualize the predictions of the signatures with Kaplan–Meier (KM) plots, we used the queue data of a published article and stratified patients according to the signature expression level. We then used the log-rank test to examine whether there was a significant difference in the OS rate between the two patient groups.

### Immunofluorescence staining

To confirm the endothelial and fibroblast subtypes in gastric tumors, we performed immunofluorescence staining for VWF and FAP in 20 pre-treatment and 20 post-treatment samples. In addition, we analyzed CD4 and CD8 to identify CD4+ T cells and CD8+ T cells by immunofluorescence staining. Serial sections (~ 4 μm) from formalin-fixed paraffin-embedded tumor tissues were stained using standard protocols. Then, we calculated the proportion of each cell type.

### Statistical analysis

All statistical analyses were performed using R. Two-sided paired or unpaired Student's t-tests and the unpaired Wilcoxon rank-sum test were used where indicated. *P* < 0.05 was considered statistically significant.

## Results

### Changes in a single-cell expression atlas of gastric cancer patients following NACT

To explore the effects on NACT in gastric cancer, we generated scRNA-seq profiles of pathologically confirmed gastric tumors by evaluating 4 samples received NACT, 4 samples without NACT and 3 pre-post treatment pairs. The clinicopathological characteristics of the patients are shown in Additional file 1: Table S1, included age, sex, tumor location, grade, Lauren classification and neoadjuvant response. After initial quality filtering, approximately 0.28 billion unique transcripts were obtained from 33,589 cells isolated. We classified these cells into clear transcriptional subtypes using clustering-distributed stochastic neighbor embedding (t-SNE) based on informative principal components (n = 21) (Additional file 3: Fig. S1A–C). Based on known classic markers, 11,535 cells (34%) were classified as epithelial cells, while the remaining cells (66%) included immune cells (i.e., myeloid, T, and B cells), endothelial cells and fibroblasts (Additional file 3: Fig. S1D).

We observed significantly fewer T cells in the post-treatment samples than in the pre-treatment samples (P = 0.010), while the other cell types showed no significant change (Additional file 3: Fig. S1E). To further verify this, we collected 40 patients with gastric cancer (20 post-treatment samples and 20 pre-treatment samples) and performed immunohistochemical staining (Additional file 3: Fig. S1F). The results showed similar downward trends in CD4+ and CD8+ T cell accumulation, indicating that NACT has important impair effects on immune cells (Additional file 3: Fig. S1G).

**Fig. 1:**
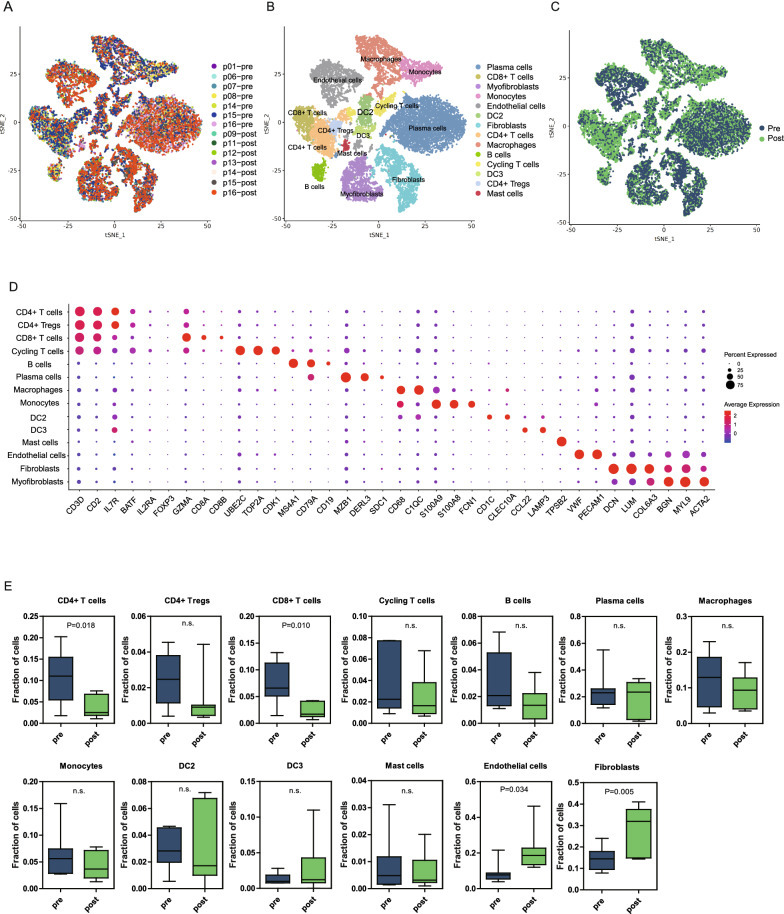
Atlas of immune cells and stromal cells in the gastric cancer TME before and after NACT. **A**–**C** t-SNE plots of all immune cells and stromal cells with each cell color coded for the patient ID (**A**), cell type (**B**), and before and after NACT (**C**). **D** Dot plot annotating the major cell types based on the cell markers in all samples. The circle size indicates the cell fraction expressing the signature at a level greater than the mean; the color indicates the mean signature expression (red, high; blue, low). **E** Fractions of different cell types in the total cell population in pretreatment (n = 7) versus post-treatment (n = 7) samples. Unpaired t test was performed to compare pretreatment and post-treatment samples. P value was shown if there was a significant difference. n.s.: not significant

Next, we performed further canonical correlation analysis on non-epithelial cells in post-treatment samples and pre-treatment samples, which revealed a complex TME that contained 14 different cell types (plasma cells, B cells, CD4+ T cells, CD4+ regulatory T (Treg) cells, CD8+ T cells, cycling T cells, dendritic cell 3 (DC 3), dendritic cell 2 (DC 2), endothelial cells, fibroblasts, myofibroblasts, mast cells, monocytes, and macrophages) (Fig. 1A–D). We observed significantly more endothelial cells and fibroblasts in the post-treatment samples than in the pre-treatment samples (*P* = 0.034 and *P* = 0.005) (Fig. 1E). In addition, the proportions of CD4+ T cells and CD8+ T cells were significantly declined during the NACT (*P* = 0.018 and *P* = 0.010). However, there was no significant change in CD4+ Tregs cells, cycling T cells, B cells, plasma cells, macrophages, monocytes, dendritic cells, and mast cells (*P* > 0.05). The detailed changes in immune cell types between pre and post NACT were showed in Additional file 2: Table S2.

### Altered expression of the nonnegative matrix factorization (NMF) 1 and NMF2 transcriptional programs improves the prognosis of patients treated with NACT

**Fig. 2:**
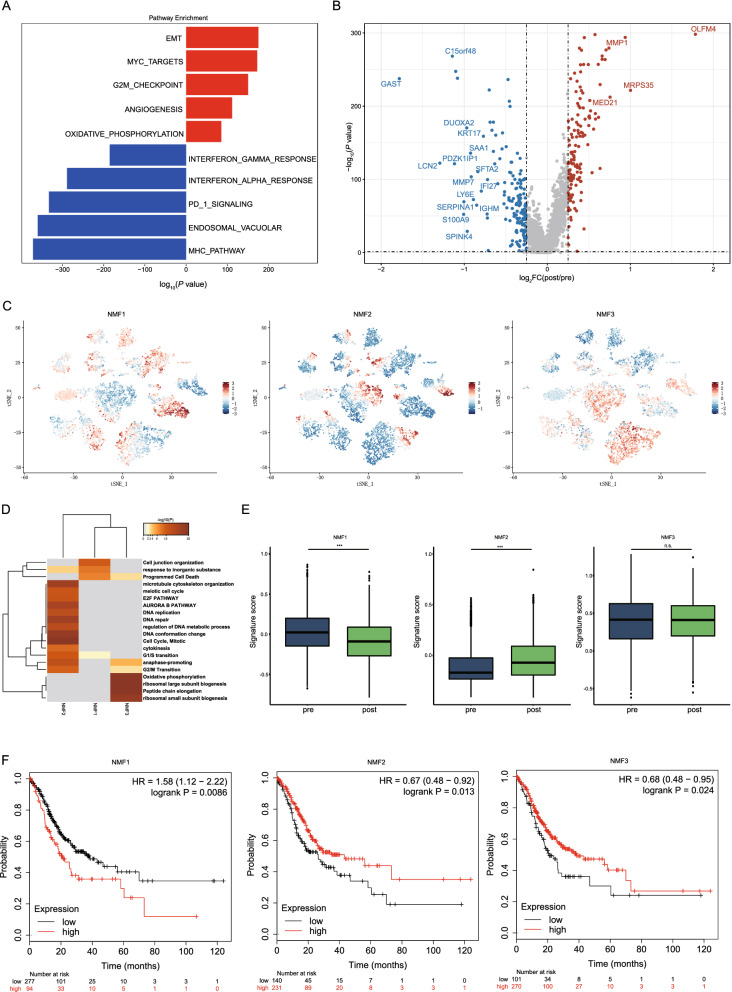
Characteristics of tumor cells before and after NACT for gastric cancer. **A** GSVA of differently expressed pathways in tumor cells before and after NACT. Two-sided unpaired limma moderated t test. **B** Volcano plot showing DEGs in tumor cells before and after treatment. **C** t-SNE plots of tumor cell signatures identified by integrative NMF. **D** Pathway enrichment of three signatures of tumor cells by Metascape. **E** Comparison of signature score of three tumor cell signatures before and after NACT. ***P < 0.001; n.s.: not significant. **F** Correlation between tumor cell signatures and prognosis. At the bottom, the number of subjects at risk were showed

To investigate how NACT affects tumor cells, we compared the pathway activation of tumor cells in all post-treatment and pre-treatment samples by gene set variation analysis (GSVA). The results showed that pathways associated with cell proliferation (MYC targets, G2M checkpoint and oxidative phosphorylation) and protumor pathways (epithelial-mesenchymal transition (EMT) and angiogenesis) were upregulated in the post-treatment samples compared with the pre-treatment samples (Fig. 2A), indicating that surviving tumor cells not killed by NACT may have higher proliferative activities and a greater ability to metastasize. The up-regulation of the oxidative phosphorylation pathway after treatment suggests that the mitochondrial functional status of tumor cells may be altered. Therefore, we analyzed the changes of mitochondrial-related genes before and after treatment, and found that a series of mitochondrial genes were up-regulated after treatment (*COX4I1*, *COX6C*, *COX7B*, *COX8A*, *NDUFA4*, *NDUFC2*, *PPA1* and *UQCRH*) (Additional file 4: Fig. S2A). These mitochondrial genes are mainly related to the ATP metabolic process and the electron transport chain [23], thus up-regulation of these genes indicates that energy metabolism of tumor cells was enhanced by NACT, which may promote the growth of tumor cells.

Moreover, immune-associated pathways (the interferon alpha (IFN-α) response, interferon gamma (INF-γ) response, PD1 signaling and MHC pathways) were downregulated after treatment (Fig. 2A), indicating that the TME developed a more immunosuppressive status. To analyze the toxicological responses of various types of cells to NACT, we simultaneously compared the changes of toxicology and drug-related pathways in tumor cells and other cell types before and after treatment. Interestingly, our results showed that the changes in these pathways were distinct for different cell types. The activities of multiple pathways (GENOTOXIC_DAMAGE, DRUG_ADME, DRUG_METABOLISM_CYTOCHROME_P4501) in tumor cells were significantly increased after treatment, while the levels of these pathways in various immune cells were down-regulated or did not change significantly after treatment (Additional file 4: Fig. S2B). These suggests that the toxic responses of different cells to NACT may be cell type-specific.

Then, to analyze changes in tumor cell gene expression between post-treatment and pre-treatment samples, we analyzed the differential gene expression of tumor cells. *IFI27*, *LCN2*, *GAST*, and *SERPINA1* were downregulated after NACT in tumor cells, while *OLFM4*, *MRPS35*, *MMP1* and *MED21* were upregulated in tumor cells (Fig. 2B). IFI27 is involved in signaling pathways of apoptosis and type-I interferon [24], while the downregulation of IFI27 may enhance the ability of tumor cells to survive. LCN2 encodes a protein of the lipocalin family, which plays roles in innate immunity by binding bacterial siderophores [25]. LNC2 is also found to be overexpressed in multiple type of cancer and is involved in tumor growth and metastasis. Gastrin, a GAST-encoded protein, is required for stomach to secret digestive enzymes and hydrochloric acid [26]. Down-regulation of GAST suggest normal function of stomach may be affected, which may be associated with adverse effects of NACT. OLFM4 is reported to be upregulated in gastric cancer [27], and is reversely correlated with NF-κB/IL-8 pathway in gastric cancer [28]. Upregulation of OLFM4 by NACT may affect NF-κB mediated immune responses. MMP1 encodes a member of matrix metalloproteinases, and was associated with cancer invasion and metastasis by activating PAR-1 [28]. In addition, MMP1 may also regulate monocyte recruitment by inducing expression of CCL-2 [29]. The roles of MRPS35 and MED21 in tumor and immune regulation are still unknown, so further research is needed to explore the mechanism for the up-regulation of these genes and their implications for treatment.

As intratumoral heterogeneity is an important feature of tumors and may play an important role in chemotherapy resistance, we next analyzed the characteristics of tumor heterogeneity in gastric tumor cells in all samples to further study the intra-tumor heterogeneity dynamics during NACT. Unsupervised nonnegative matrix factorization (NMF) analysis revealed that there were three transcriptional programs (NMF1, NMF2 and NMF3) shared among these samples (Fig. 2C). NMF1 was defined by cell junction organization markers (*CLDN4*, *ITGB1*, *JUP* and *LAMB3*) and epithelial cell differentiation markers. NMF2 involved markers of cell cycling (for example, *TOP2A*, *CDK1* and *RRM2*), and NMF3 was enriched for ribosomal genes, translation initiation and elongation factors (*EIF3E* and *EEF1A1*), and oxidative phosphorylation markers (Fig. 2D). We concluded that these programs reflected cell cycle activity (NMF2), undifferentiated progenitors (NMF3) and more differentiated epithelial cell programs (NMF1).

We further compared the difference in these three transcriptional programs between post-treatment and pre-treatment samples. The signature score of NMF1 was significantly downregulated after treatment, and gene expression analysis also showed that several cell junctions associated genes (*CLDN4*, *CTNND1*, *ITGB1* and *JUP*) were significantly down-regulated after treatment (Additional file 4: Fig. S2C). In tissues, cell junctions interconnect cells and maintain homeostasis by regulating tissue barrier function, migration and proliferation of cells [30]. Intercellular communication through cell junctions may be involved in the development of diseases [31], defects of which would induce imbalance of tissue homeostasis and are widespread in cancers. Downregulation of cell junction genes post NACT suggest that function of cell–cell junctions might be altered by NACT, which may promote tumor invasion and migration. While the NMF2 signature was significantly upregulated after treatment, the NMF3 in post-treatment samples showed comparable expression (Fig. 2E).

We then determine if these changes following NACT would affect prognosis. The downregulated NMF1 and upregulated NMF2 signatures were both associated with improved overall survival outcomes based on The Cancer Genome Atlas (TCGA) database (Fig. 2F). These data indicated that gastric cancer patients treated with NACT following gastrectomy had a better prognosis than gastric cancer patients not treated with NACT following gastrectomy.

### The changes of immune cells after NACT may be associated with immunosuppression

**Fig. 3:**
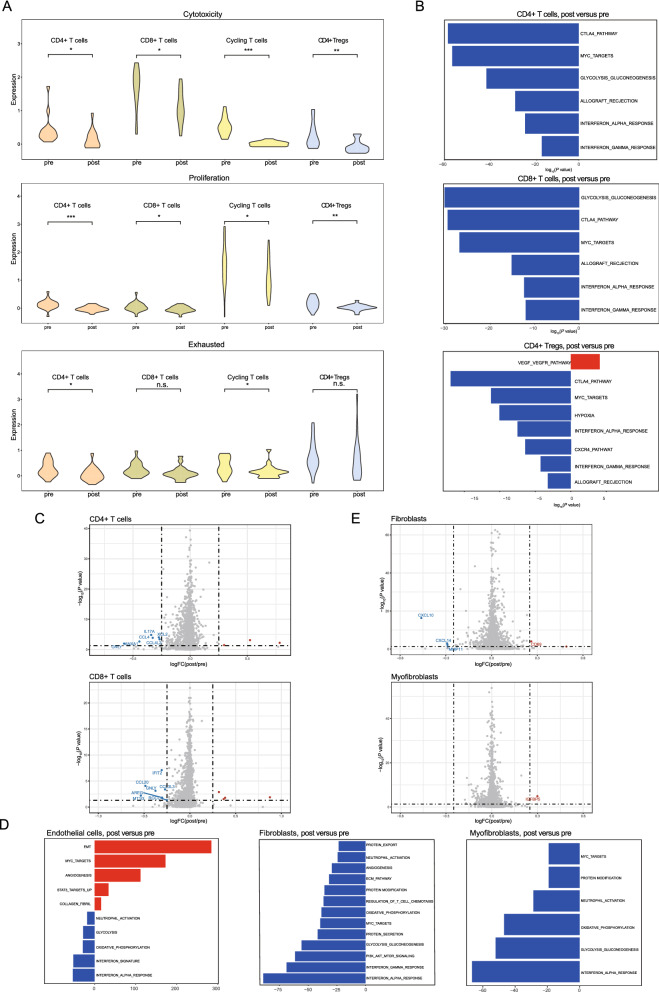
Changes in T cells and stromal cells before and after NACT. **A** Expression of cytotoxicity (top panel), proliferation (middle panel), exhaustion (bottom panel) and other T cell functional status-related gene sets in different T cell types before and after NACT. **B** GSVA of differently expressed pathways in CD4+ T cells (top panel), CD8+ T cells (middle panel), and CD4+ Tregs (bottom panel) before and after NACT. **C** Volcano plots showing DEGs in CD4+ T cells (top panel) and CD8+ T cells (bottom panel) before and after NACT. **D** GSVA of differently expressed pathways in endothelial cells (left panel), fibroblasts (middle panel), and myofibroblasts (right panel) before and after NACT. **E** Volcano plots showing DEGs in fibroblasts (top panel) and myofibroblasts (bottom panel) before and after treatment. *P < 0.05; **P < 0.01; ***P < 0.001; n.s.: not significant

As the decreased proportion of T cells indicated that chemotherapy killed immune cells, subsequently forming a more immunosuppressive environment, we further investigated the changes of pathway and gene markers among T cells. Compared to the T cells in the pre-treatment samples, all T cells in the post-treatment samples showed lower cytotoxicity and proliferative marker expression patterns (Fig. 3A, top and middle panels) than those in pre-treatment tumors. This founding has also been reported in cervical cancer after radiotherapy [16]. The exhaustion levels of CD4+ T cells and cycling T cells were significantly higher in the pre-treatment samples than in the post-treatment samples, while CD8+ T cells and CD4+ Tregs showed no significant difference (Fig. 3A, bottom panel). We observed pervasive changes in the pathway activities of T cells between the post-treatment samples and the pretreatment samples. For CD4+ T cells, CD8+ T cells and CD4+ Treg cells, multiple immune-associated pathways were downregulated, including allograft rejection, the CTLA4 pathway, and the INF-α and INF-γ responses (Fig. 3B). Allograft rejection activities in this setting are presumably related to cells showing higher reactivity to malignancy cell-encoded neoepitopes [32]. Targets of MYC, which is involved in a proliferation-related pathway, showed decreased expression after NACT treatment, which was consistent with the observed decreased T cell proportions (Figs. 1E and 3B). After NACT, T cells showed decreased expression of metal-binding and proinflammatory genes, such as *GNLY*, *ANXA1*, *CCL4*, *IL17A*, *CCL4L2* and *XCL2* in CD4+ T cells and *MT1G*, *CCL20*, *GNLY*, and *CCL3L3* in CD8+ T cells (Fig. 3C).

We further explored the characteristics of different myeloid cell subtypes comparing post-treatment versus pre-treatment samples. Monocytes and macrophages were identified based on known markers and the gene profiles of these myeloid cells were generated. Although the percentages of these cells kept stable after NACT (Fig. 1E), macrophages and monocytes exhibited varying levels of downregulation of MYC targets, the INF-α response, the INF-γ and IL6-JAK-STAT3 signaling (Additional file 5: Fig. S3A). These observations were consistent with *CXCL10* downregulation in macrophages (Additional file 5: Fig. S3B). We furtherexamined the expression of known functional signatures in macrophages and monocytes, including M1-like and M2-like signatures. Unlike pre-treatment samples, the post-treatment samples showed a decreased expression level of M1-like signature (Additional file 5: Fig. S3C).

### Stromal cells after NACT had increased angiogenesis activity

Then we investigated endothelial cells and fibroblasts at the pathway and gene level. First, post-treatment endothelial cells were shown to have MYC target upregulation (Fig. 3D, left panel), indicating that these cells were actively proliferating, which was consistent with the increased proportion of endothelial cells observed after NACT (Fig. 1E). Angiogenesis had increased activity in endothelial cells after chemotherapy (Fig. 3D, left panel), which is consistent with the function of residual tumor cells after NACT. It is well known abnormal blood vessel growth is a major factor in tumor occurrence and progression [7]. Recent evidence showed that tumor-associated endothelial cells are key players in cancer cell evasion of immune surveillance and enhanced chemoresistance [33], which was consistent with our results. Next, we examined fibroblasts, as fibroblasts are thought to be a highly plastic cell population in the TME and how fibroblast cells change with NACT treatment has not been evaluated. Compared to the pre-treatment samples, the post-treatment samples exhibited a higher percentage of fibroblast cells (Fig. 1E). Moreover, fibroblasts and myofibroblasts showed decreases in immune pathways, including the IFN-α response and IFN-γ responses (Fig. 3D, middle and right panels). *CD69* gene was highly expressed in fibroblasts in the post-treatment samples (Fig. 3E). Some chemokine-encoding genes, such as *CXCL10* and *CXCL14*, were downregulated in fibroblasts in post-treatment samples (Fig. 3E). Therefore, there is a close relationship between tumor-related fibroblasts and immune cells. Costa et al. indicated that fibroblasts contributed to immunosuppression in breast cancer [34]. Another study showed that proliferating T cells produced less IFN-γ and TNF-α when fibroblast cells were present in pancreatic cancer, thus contributing to diminished immune function [35].

### Gastric cancer is characterized by rewired cell–cell interaction networks after NACT

**Fig. 4:**
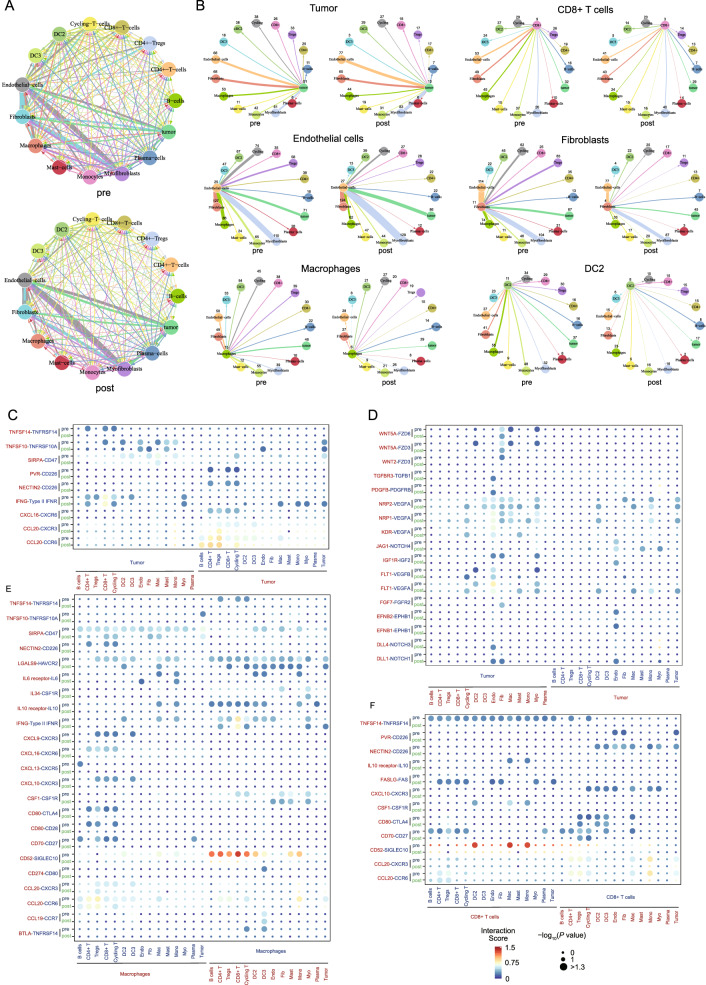
Analysis of cell–cell interactions among cells in the gastric cancer TME before and after NACT. The cell–cell communication analysis was performed using CellPhoneDB. **A** Intercellular interaction capacity between malignant cells and immune cells before (top) and after (bottom) NACT. **B** A detailed view of ligands expressed by each major cell type before and after NACT. **C**–**F** Overview of selected ligand-receptor interactions of **C** tumor cells (immune cell related ligand-receptor pairs), **D** tumor cells (stromal cell related ligand-receptor pairs), **E** macrophages, and **F** CD8+ T cells. P-values are indicated by the circle size, with the scale to the right (permutation test)

After identifying different cell subtypes present in the gastric cancer TME, we further studied the associations between different transcriptional profiles in the TME and used CellPhone DB to identify the ligand-receptor pairs and molecular interactions between the major cell types (Fig. 4A). In general, the number of predicted interactions between tumor cells and immune cells was obviously reduced after treatment, while the interactions between endothelial cells and tumor cells were enhanced (Fig. 4B and Additional file 6: Fig. S4A). These findings suggest that a new immune microenvironment balance emerged after chemotherapy.

Next, analysis of secreted ligands for the detected cognate receptors demonstrated that extensive communication occurred among different cell types in the TME (Fig. 4C–F, Additional file 6: Fig. S4B–D). For tumor cells, interactions between tumor cells and T cell subsets via CXCL16-CXCR6, CCL20-CXCR3, PVR-CD226, and NECTIN2-CD226 decreased after treatment, indicating that recruitment and co-stimulation interactions between tumor cells and T cells were reduced by treatment, which is similar with the decreased activities in recruitment and stimulatory interactions were seen for macrophages. In contrast, the interaction scores of DLL4-NOTCH3, DLL1-NOTCH1, TGFBR3-TGFB1, and IGF1R-IGF1 between tumor cells and endothelial cells increased after treatment (Fig. 4D). Communication between CD8+ T cells and other immune cells via CCL20-CXCR3, CCL20-CCR6, CD52-SIGLEC10 and NECTIN2-CD226 was decreased after treatment (Fig. 4E, F), these results indicate that T cell associated activities of activation, proliferation and recruitment declined [36, 37]. These findings were consistent with the phenomenon of CD4+ T cells, CD4+ T cells saw similar drops in activation, proliferation and recruitment.

## Discussion

In this study, we investigated the effects of NACT on tumor cells and TME in patients with gastric cancer, in order to provide the possibility for optimized following treatment therapies. We identified a total of 13 cell subtypes in 14 samples from 11 patients, detected pervasive cell state diversity at both the gene and pathway levels, and therefore generated a single-cell atlas of the stromal, epithelial, and immune cells in gastric cancer between pre-treatment and post-treatment samples.

Firstly, we found that the percentages of CD4+ and CD8+ T cells in the TME were downregulated after NACT, while no significant changes were observed in Treg cells. The important reason was that the recruitment of T cells was decreased in TME of post-treatment samples. Firstly, the connection of chemokine-chemokine receptor systems with recruiting T cells were weakened after NACT, including CXCL16-CXCR6, CCL20-CXCR3, PVR-CD226, and NECTIN2-CD226. Specifically, Eugene et al. [38] showed that TIGIT-CD226-PVR axis has important roles in various stages of T cell priming and activation. Secondly, T cells from post-treatment samples showed decreased expression of metal-binding and proinflammatory genes, such as *GNLY*, *ANXA1*, *CCL4*, *IL17A*, *CCL4L2* and *XCL2* in CD4+ T cells and *MT1G*, *CCL20*, *GNLY*, and *CCL3L3* in CD8+ T cells. *CCL4* and *CCL20* are chemoattractants which could recruit functionally distinct T lymphocyte subsets into tumors [39]. The decreases in the expression of these chemoattractants (*CCL4*, *CCL4L2*, *CCL20*, *CCL3L3*) indicate that the interactions between immune cells reduced in the treated TME. This is similar to a published study of breast cancer [40], they reported that paclitaxel impairs the expansion of immune cells, including CD4+ T cells. In addition, no significant difference observed with respect to CD4+ Tregs cells, indicating that Tregs cells may be more resistant to chemotherapeutic drugs than CD4+ and CD8+ T cells. Increasing evidence demonstrated that Tregs cells play a major role in immune escape and suppressing antitumor immune response [41].

NACT has been increasingly used to improve the curative surgical resection and decrease the risk of micrometastasis, thus prolonging the survival of gastric cancer patients. This is also confirmed in our research with the downregulated NMF1 and upregulated NMF2 signatures. Despite all this, we observed that pathways associated with cell proliferation and protumor pathways were up-regulated in the post-treatment samples compared with the pre-treatment ones, indicating the surviving tumor cells are more active. This may an important reason for NACT resistance. More efforts are still needed to study the tumor-related molecular mechanisms of oxaliplatin and 5-FU resistance. Besides the NACT, more novel treatment approaches of cancer are also concerned [42, 43].

Thirdly, we examined pathway alterations in the different cell types and found that proangiogenic pathways were activated in tumor cells and endothelial cells after NACT, indicating that angiogenic programming is a multidimensional process regulated by cancer cells and stromal cells. Neoangiogenesis is considered a prerequisite for cancer progression, and as such, inhibitors for this process have been a focus of drug development efforts [44]. Little is known on the angiogenesis response after NACT in gastric cancer on the molecular level. Marcus et al. showed that therapy-induced senescence in pancreatic cancer triggers vascular remodeling [45], which was consistent with our study of chemotherapeutic drugs. Thus, combining chemotherapy with antiangiogenic drugs might present a possible treatment strategy to improve the survival of gastric cancer patients in the future. New treatments also need to be considered, well-designed nano-antioxidants can scavenge reactive oxygen species in tumor-associated cells, presented as the downregulation of endothelial cells vascular endothelial growth factor in endothelial cells [46, 47], which was observed expanded after NACT in our study, thereby inhibiting angiogenesis and tumor growth. Additionally, combination of chemotherapy drugs with hyaluronic acid hydrogel can also achieve the targeted drug delivery and may reduce adverse reaction [48].

In addition to the cell type changes leading to an immunosuppressive environment, the endothelial cell and fibroblast percentages in post-treatment samples were upregulated compared to those in pre-treatment samples. More interactions were observed between endothelial cells and myofibroblasts in the post-treatment samples. Recent several studies showed that fibroblast cells could mediate chemotherapy resistance in several tumors through the release of paracrine signals such as cytokines, exosomes and metabolites [49–52]. For the first time, our result indicated that myofibroblasts play an important role in chemotherapy resistance in gastric cancer.

There are also some limitations to our study. First, as a small number of samples were analyzed in our study, increasing the cohort size will help to better address the influence of the NACT of TME in gastric cancer and further validate our findings. Second, spatial context we did not considered may be affected by the dissociation process, which could be addressed by dual single-cell proteomics and transcriptomics. Finally, the matched pre-treatment and post-treatment pairs would also be a better comparison, however, only 3 in 11 patients in our patient network provide these precious opportunities as clinical management difficulties remained.

## Conclusion

In conclusion, our discovery on the differences in the TME between pre-treatment and post-treatment gastric tumors provides important opportunities for the development of therapies that target these dysregulated interactions, which represents a more efficient way to inhibit Treg cells, tumor cell proliferation and invasion.

## Supplementary Information

**Additional file 1: Table S1.** Clinicopathological characteristics of the 11 patients with gastric cancer included in this study.

**Additional file 2: Table S2.** The detailed changes in immune cell types between pre and post NACT.

**Additional file 3: Figure S1.** Identification of all cells in the tumor microenvironment of gastric cancer before and after NACT. (A-C) t-SNE plots of all cells in gastric cancer samples with each cell color coded for the patient ID (A), cell type (B), and before and after NACT (C). (D) Dot plot annotating the major cell types based on the cell markers. The circle size indicates the cell fraction expressing the signature at a level greater than the mean; the color indicates the mean signature expression (red, high; blue, low). (E) Fractions of different cell types in the total cell population in pretreatment (n = 7) versus post-treatment (n = 7) samples. Unpaired t test was performed to compare pretreatment and post-treatment samples. P value was shown if there was a significant difference. n.s., not significant. (F) Immunofluorescence images of immune cell infiltration in paired pre and post NACT specimens of gastric cancer. (G) Percentage of different cell types in pretreatment (n = 20) versus post-treatment (n = 20) samples by immunohistochemical staining. Data are presented as the means ± SEM. P value was shown if there was a significant difference. n.s., not significant.

**Additional file 4: Figure S2.** The changes of pathway and genes in tumor cells before and after NAT. (A) Changes of mitochondrial-related genes before and after treatment. (B) Changes of toxicology and drug-related pathways in tumor cells and other cell types before and after treatment. (C) Changes of cell junctions associated genes before and after treatment. *P < 0.05; **P < 0.01; ***P < 0.001; n.s., not significant.

**Additional file 5: Figure S3.** Analysis of changes in the macrophages and monocytes before and after treatment. (A) Comparison of the differently expressed pathways of macrophages and monocytes before and after NACT. (B) DEG analysis of the macrophages and monocytes before and after NACT. (C) Expression of the functional signatures in macrophages and monocytes before and after NACT.

**Additional file 6: Figure S4.** Analysis of the cell–cell interactions of cells in the gastric cancer TME before and after NACT. (A) Detailed maps showing interactions between selected cell types with other cell types. (B-D) Overview of selected ligand-receptor interactions of (B) endothelial cells, (C) fibroblasts, and (D) CD4+ Tregs before and after NACT. The interaction score of interacting molecules 1 in cluster 1 and interacting molecule 2 in cluster 2 are indicated by the color.

## Data Availability

The data that support the findings of this study have been deposited into CNGB Sequence Archive (CNSA) [53] of China National GeneBank DataBase (CNGBdb) [54] with accession number CNP0001041.

## Abbreviations

CNV: Copy number variation
DC: Dendritic cell
DEGs: Differently expressed genes
EMT: Epithelial-mesenchymal transition
FC: Fold change
GSVA: Gene set variation analysis
IFN-α: Interferon alpha
INF-γ: Interferon gamma
KM: Kaplan–Meier
NACT: Neoadjuvant chemotherapy
NMF: Nonnegative matrix factorization
OS: Overall survival
scRNA-seq: Single-cell RNA sequencing
STAD: Stomach adenocarcinoma
TME: Tumor microenvironment
t-SNE: Stochastic neighbor embedding
UMIs: Unique molecular identifiers
5-FU: 5-Fluoruracil

## Code availability

R and other custom scripts for analyzing data are available upon request.
