# Supplementary figures and images for: Reconstruction of the gastric cancer microenvironment after neoadjuvant chemotherapy by longitudinal single-cell sequencing
Source: J Transl Med. 2022 Dec 6;20:563. doi: 10.1186/s12967-022-03792-y (PMC9724296; doi:10.1186/s12967-022-03792-y)

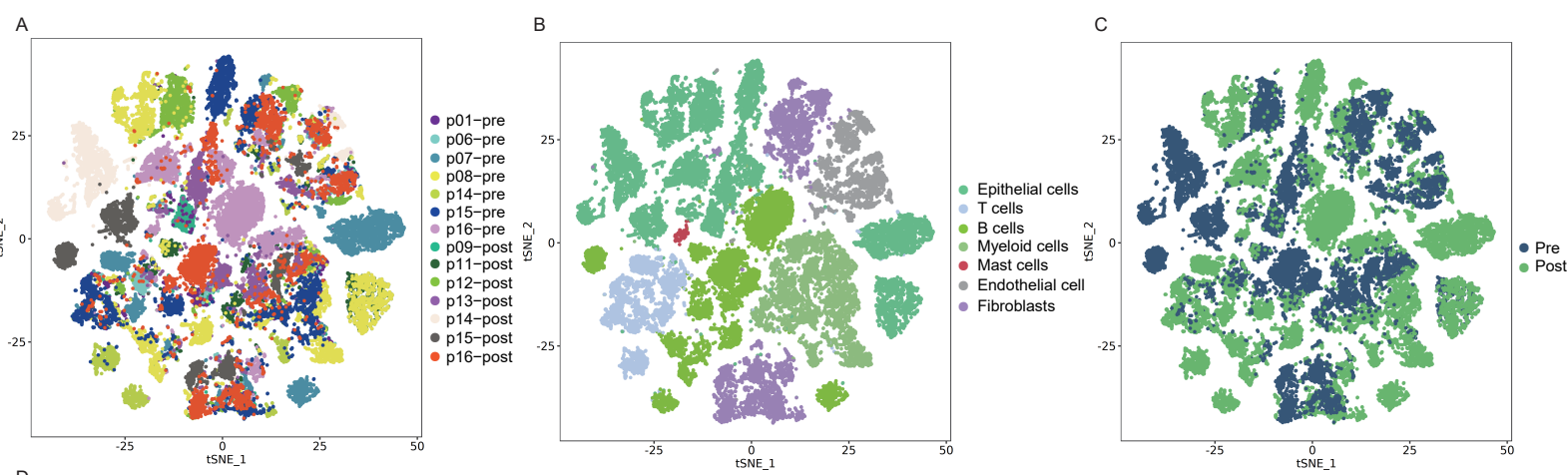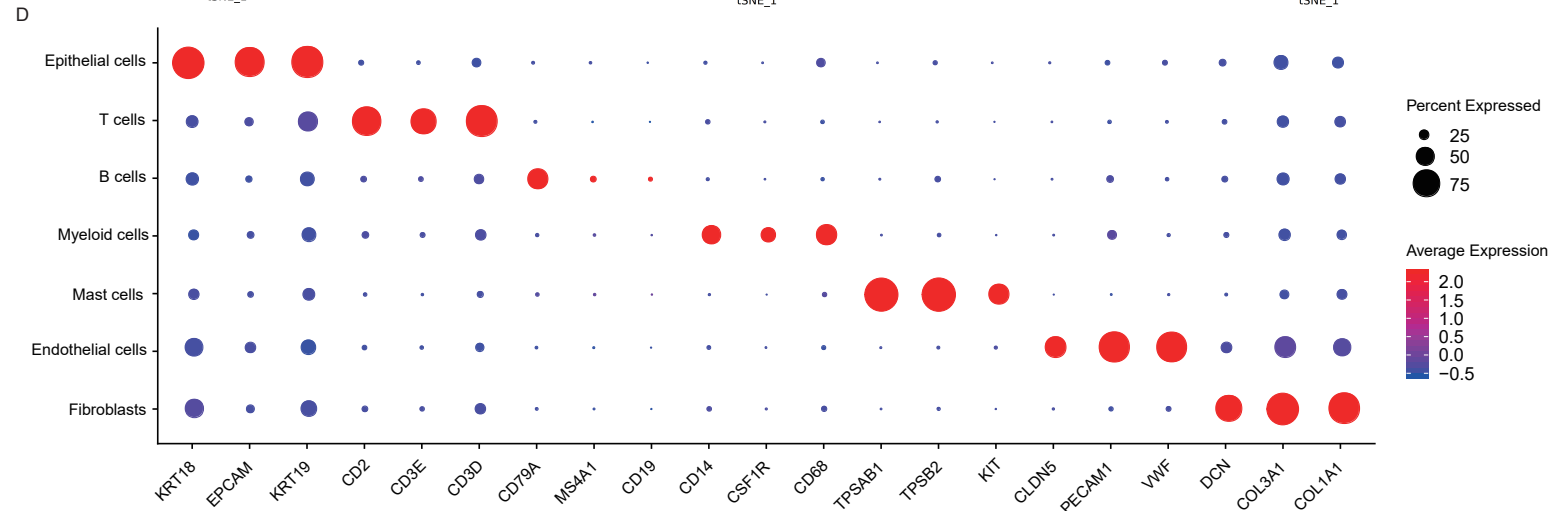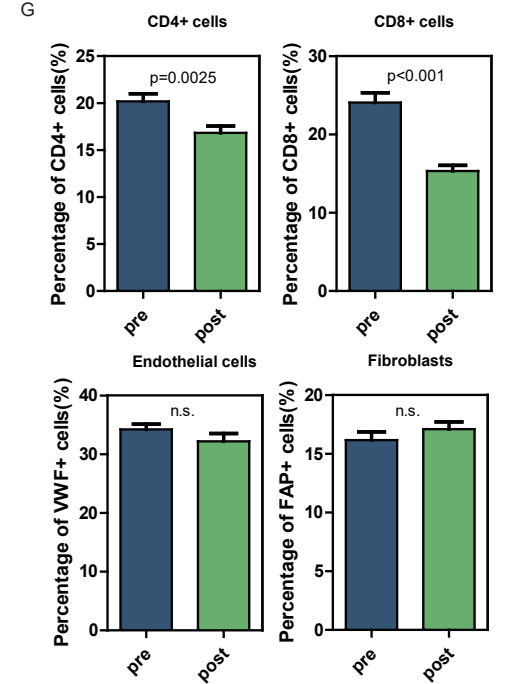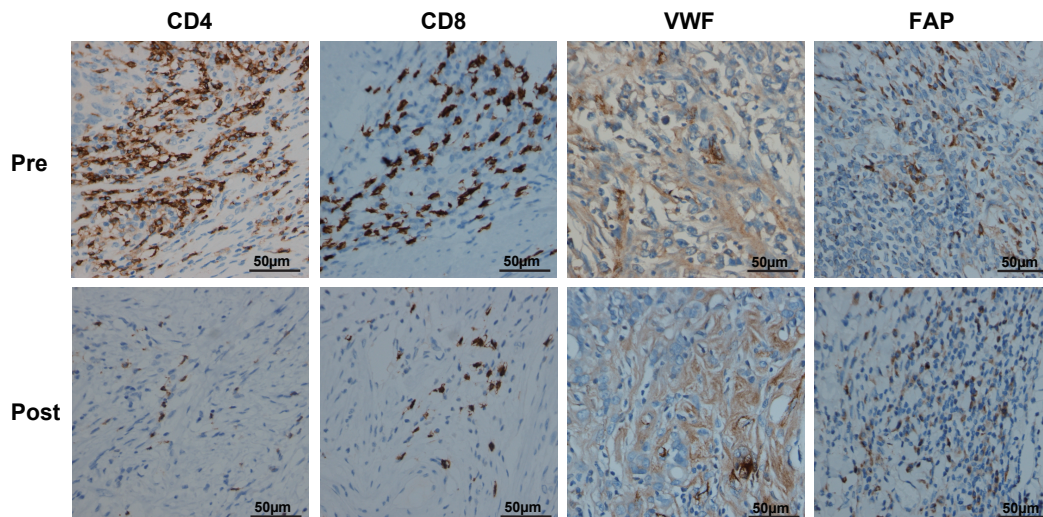

Supplement: Supplementary file 3 — Additional file 3: Figure S1. Identification of all cells in the tumor microenvironment of gastric cancer before and after NACT. (A-C) t-SNE plots of all cells in gastric cancer samples with each cell color coded for the patient ID (A), cell type (B), and before and after NACT (C). (D) Dot plot annotating the major cell types based on the cell markers. The circle size indicates the cell fraction expressing the signature at a level greater than the mean; the color indicates the mean signature expression (red, high; blue, low). (E) Fractions of different cell types in the total cell population in pretreatment (n = 7) versus post-treatment (n = 7) samples. Unpaired t test was performed to compare pretreatment and post-treatment samples. P value was shown if there was a significant difference. n.s., not significant. (F) Immunofluorescence images of immune cell infiltration in paired pre and post NACT specimens of gastric cancer. (G) Percentage of different cell types in pretreatment (n = 20) versus post-treatment (n = 20) samples by immunohistochemical staining. Data are presented as the means ± SEM. P value was shown if there was a significant difference. n.s., not significant. [file 12967_2022_3792_MOESM3_ESM.pdf]

A

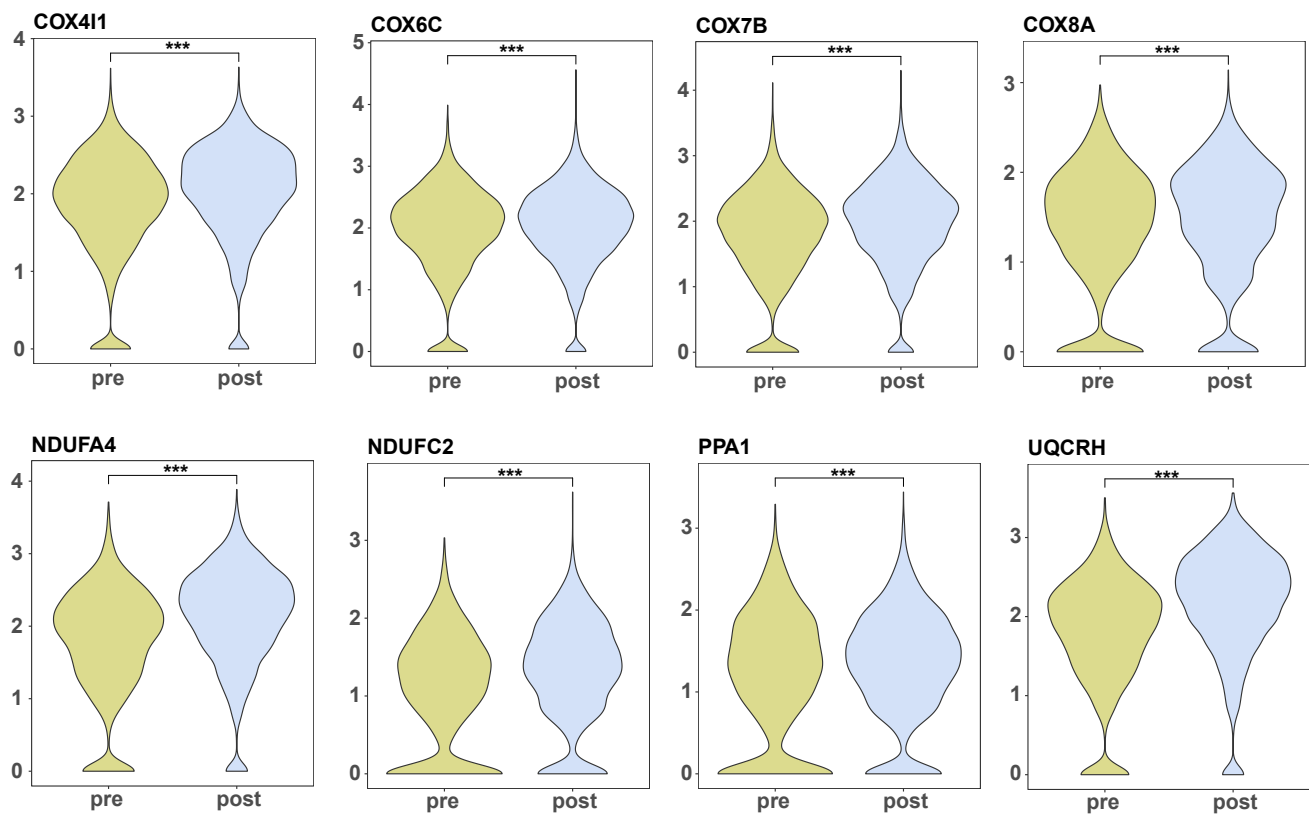

B

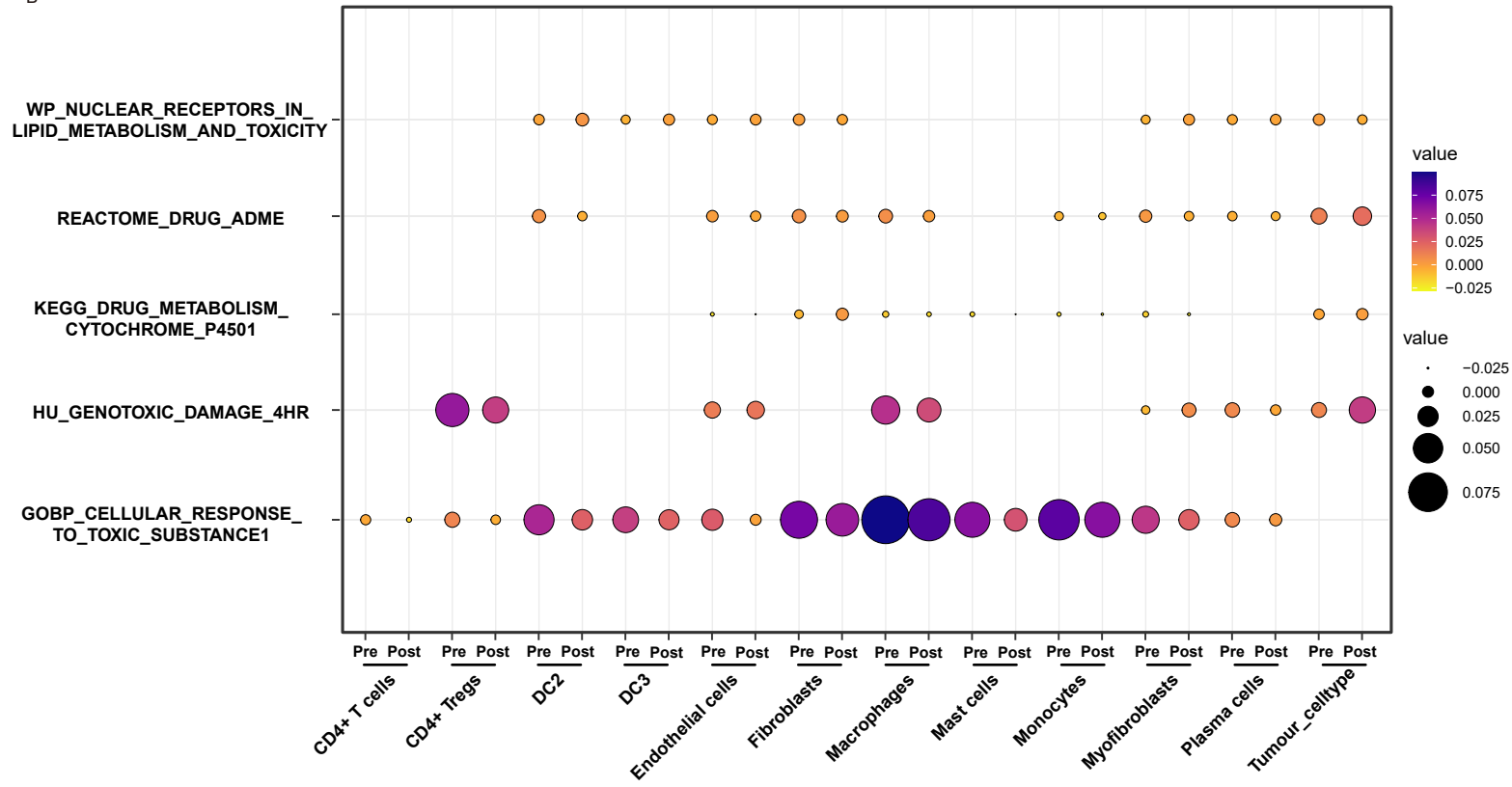

C

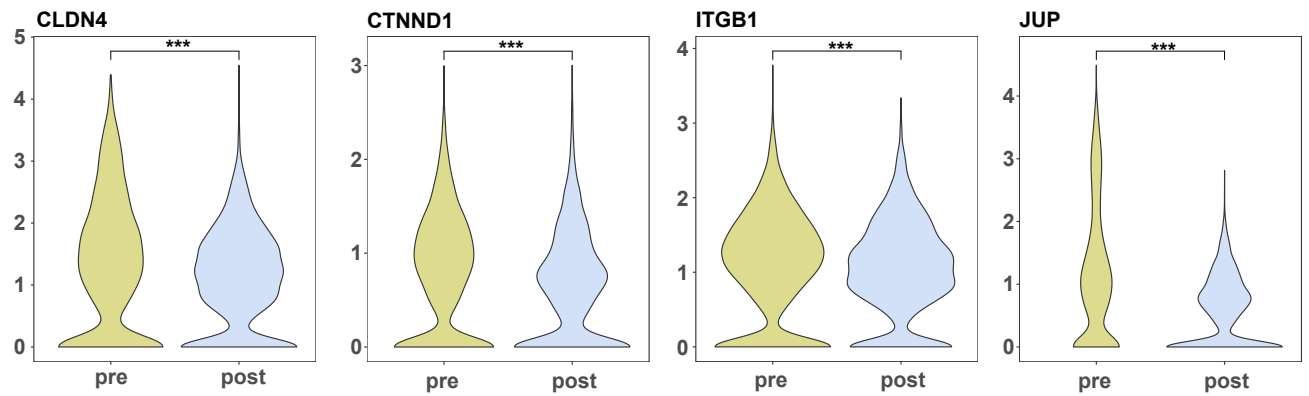

Supplement: Supplementary file 4 — Additional file 4: Figure S2. The changes of pathway and genes in tumor cells before and after NAT. (A) Changes of mitochondrial-related genes before and after treatment. (B) Changes of toxicology and drug-related pathways in tumor cells and other cell types before and after treatment. (C) Changes of cell junctions associated genes before and after treatment. *P < 0.05; **P < 0.01; ***P < 0.001; n.s., not significant. [file 12967_2022_3792_MOESM4_ESM.pdf]

A

Monocytes

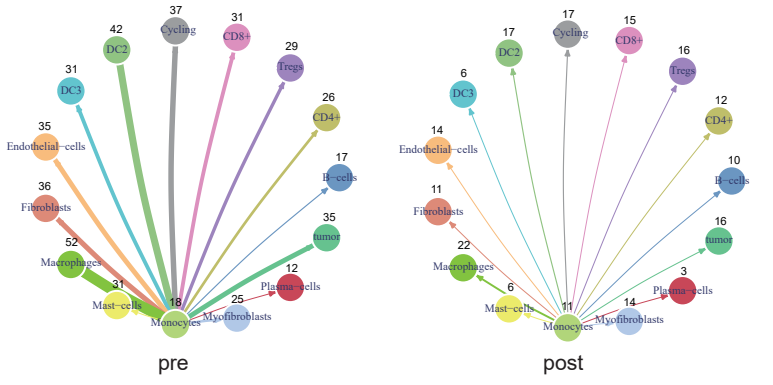

CD4+ T cells

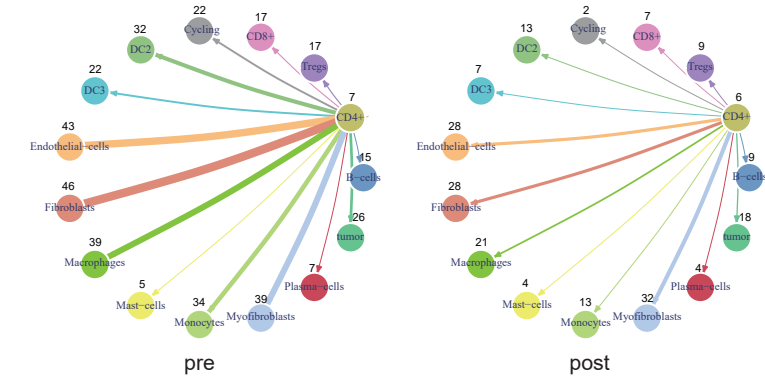

B

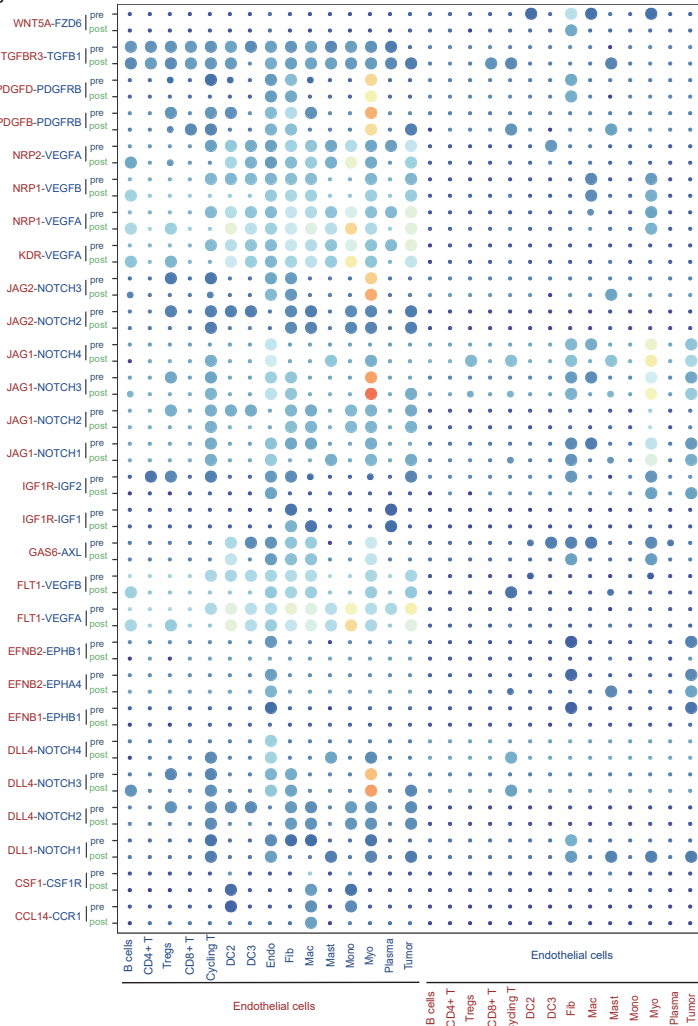

C

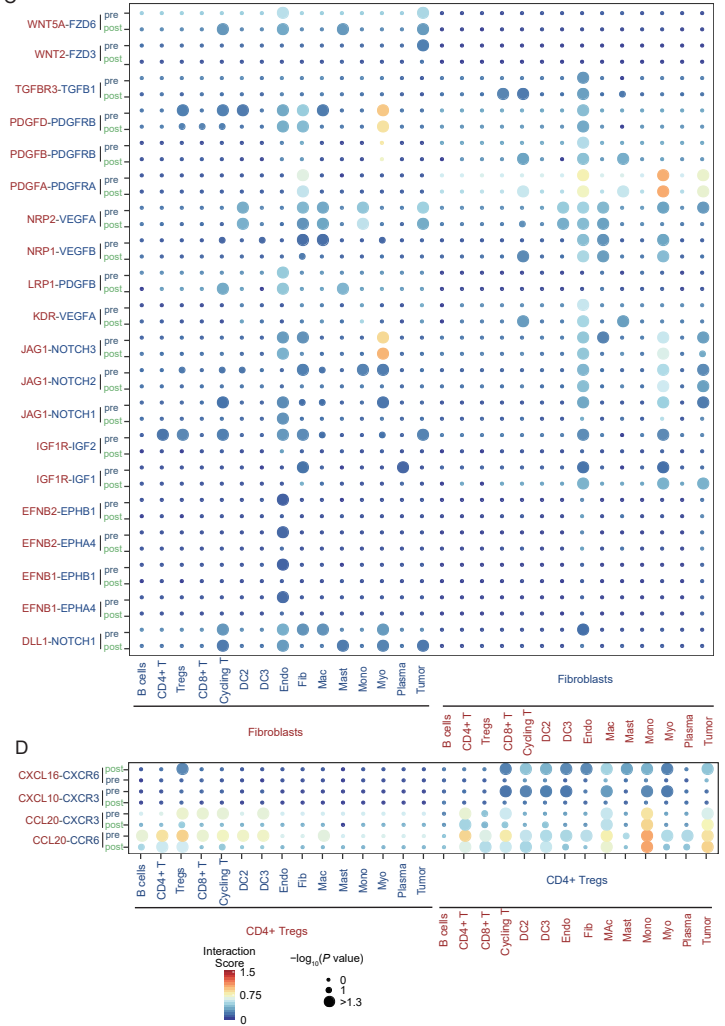

D

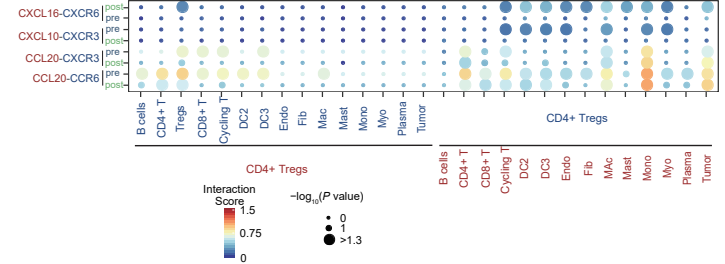

Supplement: Supplementary file 6 — Additional file 6: Figure S4. Analysis of the cell–cell interactions of cells in the gastric cancer TME before and after NACT. (A) Detailed maps showing interactions between selected cell types with other cell types. (B-D) Overview of selected ligand-receptor interactions of (B) endothelial cells, (C) fibroblasts, and (D) CD4+ Tregs before and after NACT. The interaction score of interacting molecules 1 in cluster 1 and interacting molecule 2 in cluster 2 are indicated by the color. [file 12967_2022_3792_MOESM6_ESM.pdf]
