# Supplementary material for: Reconstruction of the gastric cancer microenvironment after neoadjuvant chemotherapy by longitudinal single-cell sequencing
Source: J Transl Med. 2022 Dec 6;20:563. doi: 10.1186/s12967-022-03792-y (PMC9724296; doi:10.1186/s12967-022-03792-y)
Supplement: Supplementary file 5 — Additional file 5: Figure S3. Analysis of changes in the macrophages and monocytes before and after treatment. (A) Comparison of the differently expressed pathways of macrophages and monocytes before and after NACT. (B) DEG analysis of the macrophages and monocytes before and after NACT. (C) Expression of the functional signatures in macrophages and monocytes before and after NACT. [file 12967_2022_3792_MOESM5_ESM.pdf]

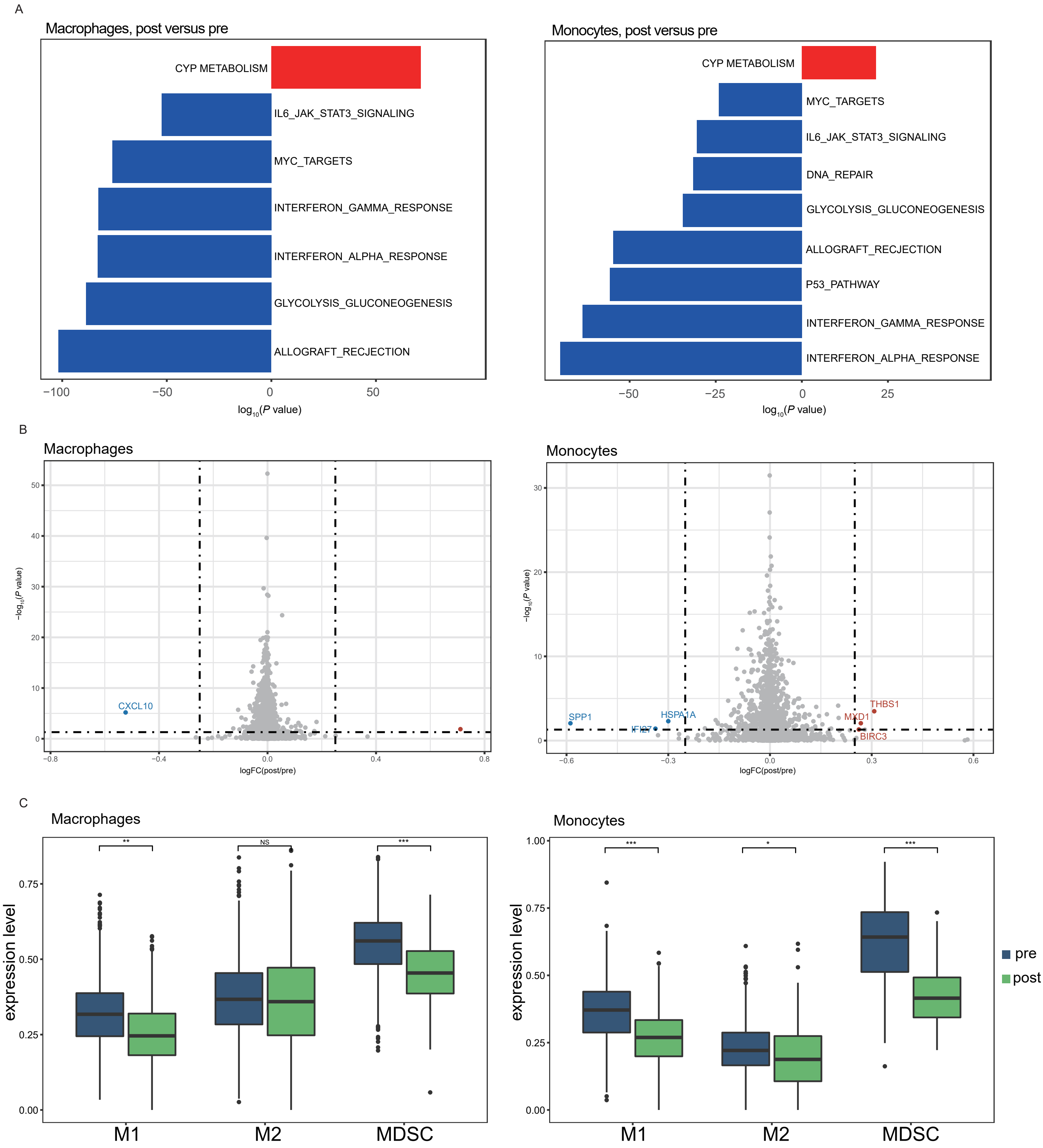
A
Macrophages, post versus pre
CYP METABOLISM
IL6_JAK_STAT3_SIGNALING
MYC_TARGETS
INTERFERON_GAMMA_RESPONSE
INTERFERON_ALPHA_RESPONSE
GLYCOLYSIS_GLUCONEOGENESIS
ALLOGRAFT_RECJECTION
−100
−50
0
50
log10(P value)
Monocytes, post versus pre
CYP METABOLISM
MYC_TARGETS
IL6_JAK_STAT3_SIGNALING
DNA_REPAIR
GLYCOLYSIS_GLUCONEOGENESIS
ALLOGRAFT_RECJECTION
P53_PATHWAY
INTERFERON_GAMMA_RESPONSE
INTERFERON_ALPHA_RESPONSE
−50
−25
0
25
log10(P value)
B
Macrophages
−log10(P value)
CXCL10
logFC(post/pre)
Monocytes
−log10(P value)
SPP1
HSPA1A
THBS1
MXD1
BIRC3
logFC(post/pre)
C
Macrophages
expression level
**
NS
***
M1
M2
MDSC
Monocytes
expression level
***
*
***
M1
M2
MDSC
pre
post
